# Use of remimazolam in living donor liver transplantation: a case report

**DOI:** 10.1186/s40981-022-00556-x

**Published:** 2022-08-22

**Authors:** Tsuguhiro Matsumoto, Kotaro Sakurai, Kazuyo Takahashi, Shuji Kawamoto

**Affiliations:** Department of Anesthesia, Kyoto University Hospital, 54, Shogoinkawahara-cho, Sakyo-ku, Kyoto, 606-8507 Japan

**Keywords:** Remimazolam, Living donor liver transplantation, Malignant hyperthermia, Hemodynamic stability

## Abstract

**Background:**

Remimazolam is an intravenous ultra-short-acting benzodiazepine with the benefit of hemodynamic stability, including blood pressure and pulse rate. We report a case in which remimazolam was used in living donor liver transplantation with stable hemodynamics.

**Case presentation:**

A 19-year-old woman underwent living donor liver transplantation due to end-stage liver disease, which is associated with a hyperdynamic state and hemodynamic instability. The patient’s sister had a history of malignant hyperthermia, so we chose total intravenous anesthesia with remimazolam. Intraoperative bleeding of seven liters occurred, but she had mild intraoperative blood pressure changes, and continuous catecholamine administration was not necessary. The patient had no memories or discomfort during the surgery.

**Conclusions:**

We maintained stable hemodynamics using remimazolam for anesthetic management of a patient undergoing a liver transplantation, which is characterized by a hyperdynamic state and circulatory instability.

## Background

Remimazolam, an ester-based, ultra-short-acting benzodiazepine is rapidly hydrolyzed by tissue esterases (mainly liver carboxylesterase) to an inactive metabolite [1, 2]. Therefore, the effects of remimazolam on lowering blood pressure are milder than propofol and this may prevent hypotension in patients with potentially unstable circulation [3]. Patients with end-stage liver disease have a hyperdynamic state in which the sympathetic nervous system becomes hypertonic and cardiac output increases as a compensatory mechanism for hypotension caused by peripheral vasodilation [4]. In this study, we report the use of remimazolam for the anesthetic management of living donor liver transplantation in a patient with end-stage liver disease who had a family history of malignant hyperthermia.

## Case presentation

A 19-year-old woman (164 cm, 74.5 kg) with a history of hilar jejunal anastomosis performed for biliary atresia at 1 month of age presented to our hospital (details of anesthesia are unknown). The previous surgery resolved the jaundice, but portal hypertension, splenomegaly, and hypersplenism progressed gradually. She had ruptured esophageal varices at the age of 9 years, and ruptured gastric varices and experienced recurrent cholangitis at the age of 18 years, leading to end-stage liver disease, which required liver transplantation. Her older sister had developed malignant hyperthermia at the time of inguinal hernia repair, with unexplained elevation of end-expiratory carbon dioxide, muscle rigidity, and elevated body temperature. Preoperative blood tests showed hemoglobin 9.7 g/dL, platelet count 21000 /μL, international normalized ratio (INR) 1.39, activated partial thromboplastin time 56.5 s, aspartate transaminase 141 U/L, alanine transaminase 62 U/L, albumin 2.3 g/dL, total bilirubin 23.3 mg/dL, direct bilirubin 16.7 mg/dL, creatine kinase 43 U/L, creatinine 0.44 mg/dL, and sodium 144 mEq/L. No hepatic encephalopathy or ascites was present, and the Child–Pugh class was B, and the MELD-Na (model for end-stage liver disease sodium) score was 22. Transthoracic echocardiography showed that cardiac function was well maintained. The patient was relatively young, and prolonged, massive doses of propofol risked inducing propofol infusion syndrome. In addition, because of the presence of multiple massive splenic artery aneurysms in the splenic hilum that could cause a critical mass of blood loss. Therefore, we chose to use total intravenous anesthesia with remimazolam. The recipient’s biological father (57 years old, 172 cm, 58.4 kg) was chosen as the donor. Since there was no history of general anesthesia and no specific assessment of susceptibility to malignant hyperthermia, general anesthesia for the donor was performed under total intravenous anesthesia with propofol. And his right lobe of the liver was removed.

**Fig. 1:**
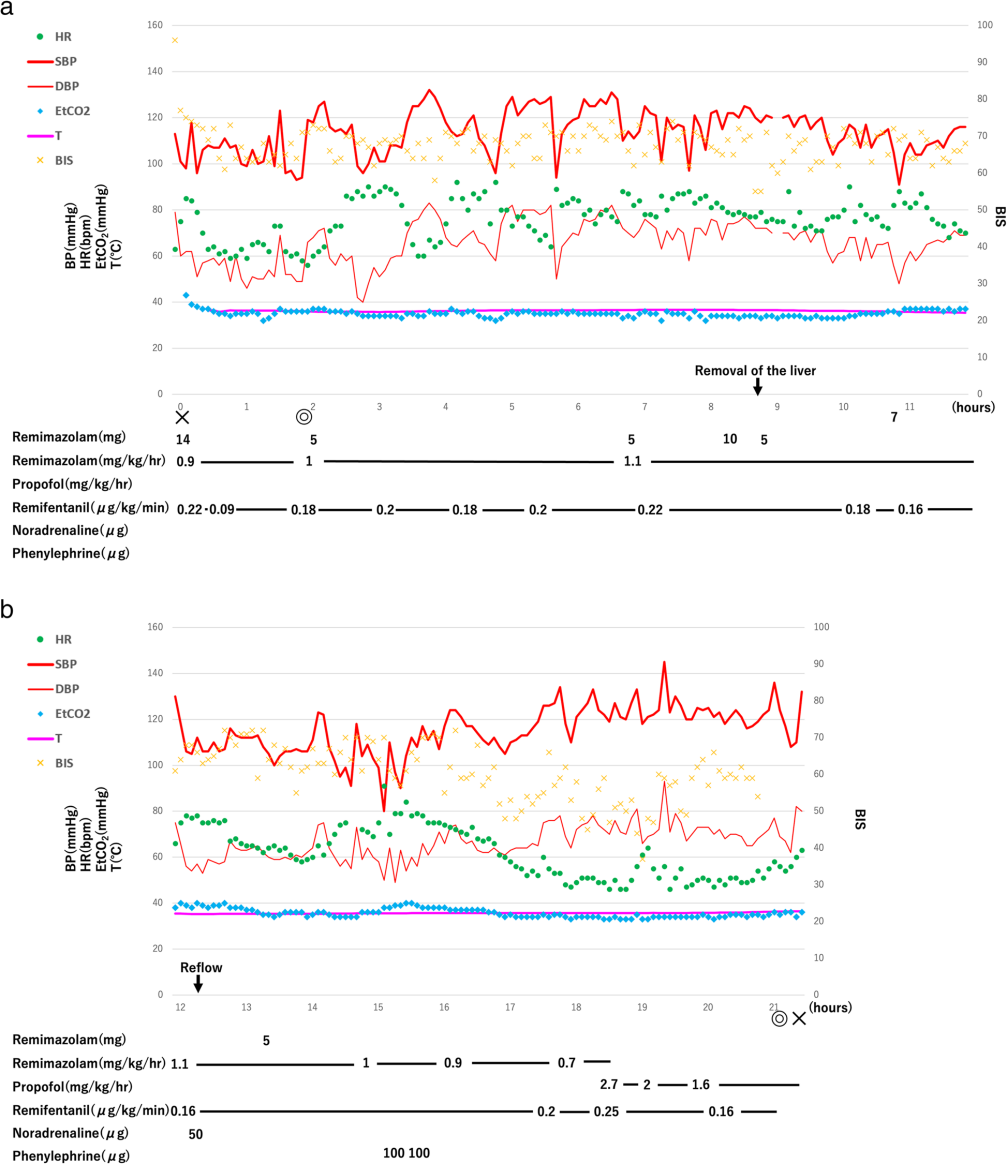
Anesthesia records. **a** The first of two anesthesia records and **b** the second. X: start of anesthesia and end of anesthesia, ◎ start of surgery and end of surgery. HR heart rate, SBP systolic blood pressure, DBP diastolic blood pressure, EtCO_2_ end tidal CO_2_, T rectal temperature, BIS bispectral index

The anesthesia records are shown in Fig. 1a, b. At induction of anesthesia, we slowly administered remimazolam 0.18 mg/kg, continuously administered remifentanil 0.22 μg/kg/min to confirm loss of consciousness and disappearance of her eyelash reflex. We also administered a bolus dose of fentanyl 150 μg and rocuronium 70 mg, 2 min before tracheal intubation and immediately started a continuous dose of remimazolam at 0.9 mg/kg/h. The bispectral index at the time of loss of consciousness was 76. Continuous intraoperative administration of rocuronium was performed, and we did not observe any body movement or electromyographic interference with the bispectral index. During surgery, we administered remimazolam and remifentanil at 0.7–1.1 mg/kg/h and 0.16–0.25 μg/kg/min, respectively. We maintained hemodynamics without the continuous administration of catecholamines. Intraoperatively, the mean arterial pressure dropped below 60 mmHg only three times. We administered only one-time bolus dose of 50 μg noradrenaline during the portal vein revascularization and two-time bolus doses of 100 μg phenylephrine during the bile duct jejunal anastomosis. We maintained the bispectral index at approximately 60–70 and adjusted the rate of continuous remimazolam administration appropriately. We treated sudden increases in the bispectral index above 70 with bolus doses of 0.06–0.1 mg/kg remimazolam. After reflow, lactate levels were largely within normal limits, and there was no evidence of progressive metabolic acidosis or hypoglycemia. Based on the above, we concluded that the grafted liver was functioning normally. The surgery and anesthesia times were 19 h, 11 min and 21 h, 50 min, respectively. Intraoperative hemorrhage was 7440 ml, and we transfused 2240 ml of red blood cells, 2400 ml of fresh frozen plasma, and 400 ml of platelet concentrate. We discontinued remimazolam and replaced it with a continuous propofol administration (1.6–2.7 mg/kg/h) at 3:30 a.m., approximately 3 h before the end of surgery. Remimazolam administration lasted for 17 h 50 min, with a total dose of 1384 mg. After surgery, we admitted the patient to the intensive care unit (ICU) still intubated. The patient rapidly awakened from anesthesia, and we extubated her at 10:30 a.m. the day after surgery because of stable respiratory and circulatory dynamics. At the 12:00 a.m. interview, the patient responded that she found herself in the ICU after surgery and that she had no memories or discomfort during the surgery. Intraoperatively and postoperatively, there were no particular signs of suspected malignant hyperthermia.

## Discussion

Remimazolam is approved in Japan for induction and maintenance of general anesthesia and is under regulatory assessment in the USA and the European Union for procedure sedation [1, 2]. Remimazolam is safe for use in patients with malignant hyperthermia at the basic research level, and there are reports of its successful clinical use [5, 6]. In the present case, no muscle biopsy was performed, but the patient’s older sister had developed malignant hyperthermia, and considering that the patient was at risk, we needed to use intravenous anesthetics to avoid inhalation agents. Both intraoperatively and postoperatively, there was no elevation of body temperature or end-expiratory carbon dioxide concentration, or masseter muscle or generalized muscular rigidity, suggestive of malignant hyperthermia during the use of remimazolam.

Compared to midazolam, remimazolam is characterized by a shorter duration of action and better adjustability [7]. Another advantage is that remimazolam can be antagonized by flumazenil. The use of remimazolam tends to achieve hemodynamic stability more easily than propofol. Therefore, remimazolam is useful for patients with potentially unstable hemodynamics [3]. Patients who undergo liver transplantation usually have end-stage liver disease and cirrhosis. In patients with cirrhosis, the metabolism of vasodilator substances is decreased, causing peripheral blood vessels to dilate and decrease vascular resistance. To compensate for this, the heart rate increases with activation of sympathetic nervous system and the renin-angiotensin system, resulting in a hyperdynamic state [4]. The response to heart rate changes in intravascular volume, such as bleeding, is small, and blood pressure can easily decrease. In addition, portal hypertension causes splenic hypertension, which leads to anemia and low platelet counts. In combination with decreased production of coagulation factors, bleeding during transplant may be massive. Moreover, severe hypotension and arrhythmias may arise after liver graft reperfusion. In addition, liver transplantation procedures involve repeated clamping and unclamping of the portal vein, collateral blood vessels, and the inferior vena cava. These factors cause circulatory instability during the anesthetic management of liver transplantation [4]. In this case, the decrease in blood pressures were extremely mild, and the required use of vasoconstrictors was minimal. The patient’s young age and normal cardiac function may have contributed to this. However, remimazolam may have contributed to hemodynamic stability.

Remimazolam is metabolized by tissue carboxylesterase, and its clearance is decreased in patients with severe hepatic impairment. Therefore, careful administration is required [8]. In patients with severe hepatic impairment, clearance was 38.1% lower than in healthy volunteers in the three-compartment recirculation model, and thus, recovery was slightly delayed (healthy: 8.0 min, moderate: 12.1 min, severely hepatic impairment: 16.7 min) [8]. Furthermore, the volume of distribution of remimazolam in patients with severe liver failure (Child–Pugh class C) is 1.01 L/kg [1]. The protein binding rate is approximately 92% [1]. In this study, the patient had a significant hepatic damage, and a long anhepatic period between the removal of the liver and the reperfusion of the transplanted liver. This might have caused the prolonged metabolism of remimazolam. In addition, rapid bleeding could increase blood levels of remimazolam. We adjusted the dose of remimazolam from 0.9 to 1.1 mg/kg/h depending on the vital signs, the bispectral index, and electroencephalogram findings. After reperfusion and stabilization of vital signs, we discontinued remimazolam and started propofol approximately 3 h before the end of the surgery. We extubated her the next day without the use of flumazenil, suggesting that there was no apparent prolongation of the effects of remimazolam. The electroencephalogram changes during remimazolam infusion include an initial increase in beta frequency band and a late increase in delta frequency band [9]. The bispectral index increased suddenly several times during the operation. However, although the patient was not evaluated in detail using the modified Brice questionnaire, no intraoperative awareness was noted in the interview the day after surgery.

In summary, we maintained stable hemodynamics using remimazolam for anesthetic management of a patient undergoing a liver transplantation, which was characterized by a hyperdynamic state and circulatory instability.

## Data Availability

All data generated or analyzed in this study are included in this article.

## Abbreviations

ICU: Intensive care unit
MELD-Na: Model for end-stage liver disease sodium
